# McArdle disease does not affect skeletal muscle fibre type profiles in humans

**DOI:** 10.1242/bio.20149548

**Published:** 2014-11-28

**Authors:** Tertius Abraham Kohn, Timothy David Noakes, Dale Elizabeth Rae, Juan Carlos Rubio, Alfredo Santalla, Gisela Nogales-Gadea, Tomas Pinós, Miguel A. Martín, Joaquin Arenas, Alejandro Lucia

**Affiliations:** 1 UCT/MRC Research Unit for Exercise Science and Sports Medicine, Department of Human Biology, University of Cape Town, PO Box 115, Newlands 7725, South Africa; 2 Mitochondrial and Neuromuscular Diseases Laboratory, i+12 Research Institute, Hospital 12 de Octubre, 28041 Madrid, Spain; 3 Department of Sport Science, Universidad Pablo de Olavide, 41013 Seville, Spain; 4 Neuromuscular Diseases Unit, Institut de Recerca del Hospital de la Santa Creu i Sant Pau, Universitat Autónoma de Barcelona, 08193 Barcelona, Spain; 5 Departament de Patologia Mitocondrial i Neuromuscular, Hospital Universitari Vall d'Hebron, Institut de Recerca (VHIR), Universitat Autónoma de Barcelona, 08193 Barcelona, Spain; 6 European University of Madrid, 28670 Madrid, Spain

**Keywords:** Myosin heavy chain, Glycogen storage disease V, Phosphorylase deficiency

## Abstract

Patients suffering from glycogen storage disease V (McArdle disease) were shown to have higher surface electrical activity in their skeletal muscles when exercising at the same intensity as their healthy counterparts, indicating more muscle fibre recruitment. To explain this phenomenon, this study investigated whether muscle fibre type is shifted towards a predominance in type I fibres as a consequence of the disease. Muscle biopsies from the *Biceps brachii* (BB) (n = 9) or *Vastus lateralis* (VL) (n = 8) were collected over a 13-year period from male and female patients diagnosed with McArdle disease, analysed for myosin heavy chain (MHC) isoform content using SDS-PAGE, and compared to healthy controls (BB: n = 3; VL: n = 10). All three isoforms were expressed and no difference in isoform expression in VL was found between the McArdle patients and healthy controls (MHC I: 33±19% vs. 43±7%; MHC IIa: 52±9% vs. 40±7%; MHC IIx: 15±18% vs. 17±9%). Similarly, the BB isoform content was also not different between the two groups (MHC I: 33±14% vs. 30±11%; MHC IIa: 46±17% vs. 39±5%; MHC IIx: 21±13% vs. 31±14%). In conclusion, fibre type distribution does not seem to explain the higher surface EMG in McArdle patients. Future studies need to investigate muscle fibre size and contractility of McArdle patients.

## INTRODUCTION

McArdle patients suffer from glycogen storage disease V and have limited endogenous glycogen for use during exercise. They find exercise difficult and fatigue quickly, presumably since they initially rely on blood glucose from exogenous sources for fuel. They often, however, report a “second-wind” effect, coinciding with the mobilisation of free-fatty acids for fuel (Lucia et al., 2008; van Adel and Tarnopolsky, 2009). As a result, many McArdle patients avoid exercise, or are advised that exercise is contra-indicated for their condition, which may inadvertently decrease their quality of life. A recent study found that patients suffering from McArdle disease produced less force, fatigued faster, and surprisingly recorded almost three times as much surface electrical activity in their skeletal muscles (measured by electromyography) compared to healthy controls exercising at the same intensity (Rae et al., 2010). Surface EMG activity correlates well with the number of active motor units (Moritani and Muro, 1987), and are therefore good proxies for muscle fibre recruitment. Although some evidence exist that muscle fibre type correlates with EMG activity *in vitro*, it is still unclear whether this holds true *in vivo* (Farina, 2008; Kupa et al., 1995; von Tscharner and Nigg, 2008). In theory, this should hold true, but current techniques in pinpointing EMG activities to a particular fibre or fibre type is still in its infancy. Nevertheless, Rae et al. (Rae et al., 2010) suggested that this excessive muscle recruitment might be attributed to altered contractile properties of the diseased muscle, perhaps compounding the exercise intolerance experienced by McArdle patients.

Muscle fibre type plays an important role in determining the amount of force and power output a muscle can produce, as well as its resistance to fatigue. It is well documented that type I fibres produce less force and power, have slower contraction speeds, but are more resistant to fatigue than type IIA muscle fibres (Essén-Gustavsson and Henriksson, 1984; Kohn and Noakes, 2013). The fatigue resistance is primarily attributed to abundant mitochondria found in type I fibres and its ability to utilise fat as a fuel source to generate ATP for muscle contraction (Bottinelli, 2001; Pette, 1985). Type IIA fibres produce force and power levels below that of type IIX fibres, but significantly higher than type I fibres, have high mitochondrial content, rely on glycogen and fat stores for ATP replenishment, and withstand fatigue for longer periods of time than type IIX fibres (Pette, 1985). It is well known that endurance athletes have predominantly type I and IIA fibres in their *Vastus lateralis* (VL) muscle, with high mitochondrial numbers (Essén-Gustavsson and Henriksson, 1984; Kohn et al., 2007b). Sedentary individuals and sprinters have a greater proportion of type IIX muscle fibres, but rarely more than their type I or type IIA fibres (Andersen et al., 1994; Kohn et al., 2007a). Diseases, such as Duchene's muscular dystrophy, was shown to shift the fibre type profile to type I and IIA, even when they also struggle to perform exercise (Fink et al., 1990). To date, very little data exist surrounding muscle fibre type of McArdle patients, specifically whether this disease affects overall fibre type composition.

This study therefore aimed to determine muscle fibre type composition in biopsies from the *Biceps brachii* (BB) or the VL from McArdle patients, and compare it to healthy controls. The hypothesis was that McArdle patients would have a predominance of type I muscle fibres and significantly more than their healthy counterparts, resulting from an adaptation to rely primarily on fat as a fuel source, due to their inability to utilise their glycogen stores.

## RESULTS AND DISCUSSION

To our knowledge, this is the first study reporting the muscle fibre type composition in patients with McArdle disease. The main finding is that McArdle patients had similar fibre type profiles as controls.

**Fig. 1.**
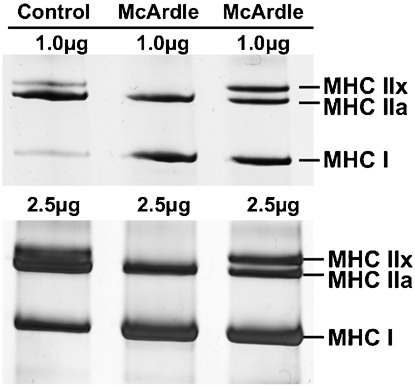
Separation of the myosin heavy chain (MHC) isoforms into three distinct bands using SDS-PAGE of a control and two McArdle patients. In each well, a total of 1 µg protein and 2.5 µg protein were loaded in the top and bottom gels, respectively.

Three MHC isoforms (I, IIa and IIx) were found in the McArdle and control samples. The MHC isoforms were separated and identified in healthy control samples using the SDS-PAGE technique and corresponded to (top to bottom) MHC IIx, MHC IIa and MHC I (Fig. 1). This method is well characterised and provides good estimates of relative muscle fibre type from biopsies (Andersen et al., 1994; Curry et al., 2012; Kohn et al., 2007b). No additional bands were detected in control and McArdle samples that may indicate the expression of the IIb, neonatal or embryonic isoforms, as is found in low quantities in regenerating fibres due to exercise or disease (Bottinelli, 2001; D'Antona et al., 2003; Fink et al., 1990; Schiaffino and Reggiani, 1996; Williams et al., 1993).

As McArdle disease is rare [∼1 in 170 000 people in Spain (Lucia et al., 2012)], the patient's data and biopsies were collected over a 13-year period, from both males and females, with a wide age range (17 to 76 years) and biopsies were taken non-intentionally from two muscle groups (VL and BB). Given that the data had to be grouped by muscle for analysis, the groups are heterogeneous, the sample size relatively small and thus interpretation limited.

**Fig. 2.**
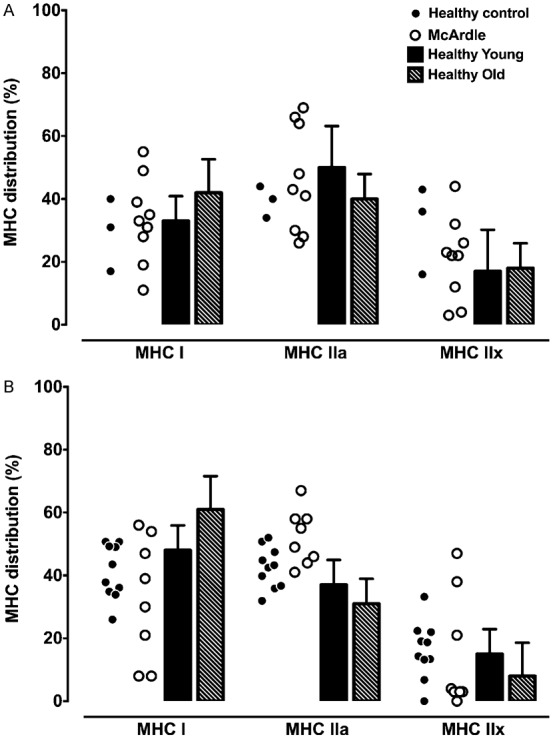
Myosin heavy chain isoform distribution in (A) *Biceps brachii* and (B) *Vastus lateralis* from healthy participants (closed circles) and patients diagnosed with McArdle disease (open circles). Values from the literature (bars) are included for both muscle groups as reference points (Klitgaard et al., 1990b). Where appropriate, data are presented as mean ± SD.

The MHC isoform content of VL muscle of McArdle patients and healthy controls were not different (MHC I: 33 ± 19% vs. 41 ± 9%; MHC IIa: 52 ± 9% vs. 43 ± 7%; MHC IIx: 15 ± 18% vs. 16 ± 9%) and was also the case for the BB muscle (MHC I: 33 ± 14% vs. 29 ± 11%; MHC IIa: 46 ± 17% vs. 39 ± 5%; MHC IIx: 21 ± 13% vs. 32 ± 14%) (Fig. 2). The relative fibre type distributions obtained in this study are well in range with published data on sedentary and recreationally active individuals. For young (i.e. 20 to 30 years of age), inactive to recreationally active individuals, the range in fibre type distribution in the VL is approximately between 35 and 60% MHC I and 35 to 50% MHC IIa. The amount of MHC IIx expressed in this muscle is not particularly high, and can constitute approximately 25% of the total MHC isoform composition (Klitgaard et al., 1990b; Kohn et al., 2011; Monemi et al., 1999). Previous research has found a weak, yet significant positive correlation between the percentage type I muscle fibres and age (Gouzi et al., 2013). Unfortunately, with the large range in standard deviation and overlap between the 19 studies evaluated in the study by Gouzi et al. (Gouzi et al., 2013), the average type I profile of the elderly ranged from 45 to 55%. It therefore suggests that age has a less important role in determining fibre type. In the present study, there was no relationship found between any of the MHC isoforms from the two muscle groups and age.

It is well known that endurance athletes have a predominance in MHC I and IIa isoforms and very little to no MHC IIx in their VL, whereas resistance athletes may present with values similar to inactive individuals (D'Antona et al., 2006; Kohn et al., 2007b). Thus, physical activity levels seem to play a greater role in determining the fibre type profile of a muscle. The MHC isoform content of the BB from the controls and McArdle patients were similar in range and agreed with data from Klitgaard et al. (Klitgaard et al., 1990a) (included in Fig. 2), Monemi et al. (Monemi et al., 1999) and Elder et al. (Elder et al., 1982). It has to be emphasised though, that gender, age and trained status seems to play a greater role in determining the size of the muscle fibre. The McArdle patients of this study were encouraged by their respective physicians to refrain from physical activity except for those necessary for daily living. The patients were therefore untrained and sedentary (Maté-Muñoz et al., 2007).

Interestingly, isoform profiles for the McArdle patients were similar for both muscles analysed, with the MHC I and IIa being almost double that of MHC IIx. In contrast, the BB of controls tended to have an equal distribution between the three isoforms, whereas the VL predominantly expressed MHC I and IIa.

Unfortunately, the biopsy samples from this study could not be used to determine cross-sectional area of individual muscle fibres due to the long-term storage thereof. This would have been useful to help account for the higher EMG activity at the same workload observed previously (Rae et al., 2010). However, it seems that fibre diameter of only type I fibres are affected by McArdle disease, leaving the type II fibres unchanged (Felice et al., 1996). In contrast, patients with Duchene's muscular dystrophy have a predominance of type I and IIA fibres, with very few to no type IIX fibres (Fink et al., 1990). Thus, it could be argued that, because fibre type in McArdle patients is not affected, and neither is the type II fibre diameters, as proposed by previous research, the contractility of individual fibres may be affected. It is well known that contractility is affected by fibre diameter and the latter affected by exercise type (Bottinelli, 2001). For example, resistance training increases muscle fibre diameters, but normalised force stays the same in single fibres (Shoepe et al., 2003). Endurance exercise, on the other hand, tends to decrease fibre size, but normalised force increases after training (Trappe et al., 2006). Other factors such as myofibrillar concentration (i.e. myosin and actin), myosin light chains and Ca^2+^ sensitivity could affect the performance of single fibres (Bottinelli, 2001; Ottenheijm et al., 2011).

Limitations of the present study are: (i) small sample size for the control BB muscle (n  =  3) and overall power of the statistical analyses, (ii) unknown biopsy location and depth for certain individuals, (iii) no histology available when analyses were performed.

With regards to the small sample size, this study was unable to obtain more biopsies from healthy individuals from the BB. As this muscle is considerably smaller than the VL, the norm today is to perform most biopsies from the latter. Therefore, a limited number of studies exist in the current literature concerning biopsies from the BB. For both muscle groups, the sample sizes were low, and therefore affected the power of the statistical analyses. Using only the MHC I of the VL (percentage ± SD, n), the overall power was calculated as 0.195 with an alpha of 0.05. By using the same data but increasing the samples size to 33 per group, the power only increased to 0.213. To obtain a significant power (0.90), we calculated the number of participants in each group and amounted to 79 subjects per group, which is an unrealistic number in terms of data collection.

It is well known that the location and depth of the biopsy can significantly influence the fibre type composition (Elder et al., 1982; Kohn and Myburgh, 2007). Unfortunately, it is not clear where the exact location of the biopsy sites were for the McArdle patients. As most neurologists prefer the open biopsy technique, it was assumed that the McArdle biopsies (VL and BB) and the BB from the three controls were performed using the open biopsy technique, which will mostly sample superficially. On the other hand, the control VL biopsies were all obtained using the percutaneous suction assisted technique at a standard depth of 2 cm. Nevertheless, the data from the present study for VL and BB falls well within those obtained for the control participants, as well as the current literature. If the proposed hypothesis were true, a predominance in type I fibres would have been observed in McArdle patients, irrespective of biopsy location.

In conclusion, the data from this study suggested that fibre type distribution does not explain the higher surface EMG in McArdle patients. The skeletal muscle groups (VL and BB) of the male and female McArdle patients used in this study expresses a normal range for each MHC isoform compared to healthy individuals. These data support the suggestion that altered single muscle fibre contractility, fibre diameters or other proteins forming part of the contractile apparatus may partly account for the abnormal muscle recruitment previously observed in McArdle patients (Rae et al., 2010).

## MATERIALS AND METHODS

### Participants

Muscle samples from McArdle patients analysed in this study were collected as part of a larger study, the details of which have been published previously (Maté-Muñoz et al., 2007). Written informed consent was obtained from these patients that a part of the tissue could be used for research purposes and was approved by the Ethics Committee (Hospital 12 de Octubre, Madrid, Spain). Muscle biopsies were collected from 17 patients (8 male, 9 female, age: 41 ± 18 years) diagnosed with McArdle disease over a 13-year period. VL biopsies (3 male, 5 female, mean age: 48 ± 19 years) or BB biopsies (5 male, 4 female, mean age: 35 ± 16 years) were rapidly frozen in liquid nitrogen and stored at −80°C. VL biopsies from 10 recreationally active males (mean age: 25 ± 2) and BB biopsies from two males and one female (age: 35 ± 10), forming part of previous studies, served as healthy control. Descriptive statistics of the participants are listed in Table 1.

**Table 1.**
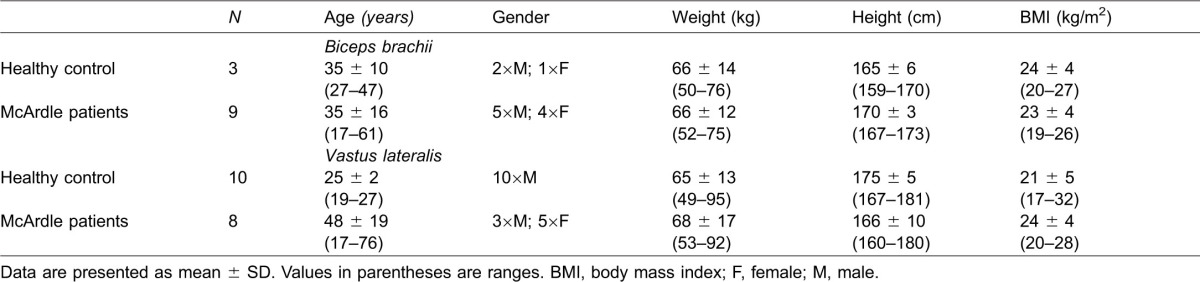
Descriptive data of healthy control participants and McArdle patients

McArdle disease was diagnosed by genetic analysis and, in most cases, also confirmed by undetectable myophosphorylase activity and negative myophosphorylase histochemical staining in the muscle biopsies, as previously published (Lucia et al., 2008; Lucia et al., 2012).

### Relative muscle fibre type quantification

The myosin heavy chain isoform content of the muscle samples was separated using SDS-PAGE, as described in detail (Kohn et al., 2011). Electrophoresis was carried out at 70 V for 4 hours at 4°C, followed by 275 V for 20 hours. Gels were silver stained, scanned and the densitometric profile of the bands determined (Fig. 1) (Un-Scan-It, Silk Scientific Inc, Utah). Each sample was twice electrophoresed, the first at a total concentration of 1 µg and the second at 2.5 µg to ensure adequate detection of the three isoforms and any additional bands that could correspond to the embryonic, neonatal or the MHC IIb isoform.

### Statistical analyses

Data are presented as mean ± standard deviation and subjected to a non-parametric ANOVA (Kruskal-Wallis test) and a Dunn's multiple comparisons post-hoc test (GraphPad Prism). Relationships were determined using the non-parametric Spearman r correlation coefficient. Significance was set at *P* < 0.05.

### List of abbreviations

BB, Biceps brachii; EMG, Electromyographic; MHC, Myosin heavy chain; VL, Vastus lateralis.
